# Regulatory T Cell Phenotype Related to Cytokine Expression Patterns in Post‐COVID‐19 Pulmonary Fibrosis and Idiopathic Pulmonary Fibrosis

**DOI:** 10.1002/iid3.70123

**Published:** 2025-01-14

**Authors:** Sara Gangi, Laura Bergantini, Irene Paggi, Marco Spalletti, Paolo Cameli, Elena Bargagli, Miriana d'Alessandro

**Affiliations:** ^1^ Department of Medical and Surgical Sciences & Neurosciences, Respiratory Diseases Unit Siena University Hospital Siena Tuscany Italy

**Keywords:** idiopathic pulmonary fibrosis, pro‐fibrotic cytokines, pulmonary fibrosis post‐COVID19, regulatory T cells

## Abstract

**Background:**

Post‐coronavirus disease 19 lung fibrosis (PCLF) shares common immunological abnormalities with idiopathic pulmonary fibrosis (IPF), characterized by an unbalanced cytokine profile being associated with the development of lung fibrosis. The aim of the present study was to analyze and compare the different subsets of CD4‐ and CD8‐T cells, along with specific cytokine expression patterns, in peripheral blood (PB) from patients affected by PCLF and IPF and healthy controls (HCs).

**Methods:**

One‐hundred patients followed at the Rare Lung Disease Center of Siena University Hospital were enrolled. Eight HCs were recruited. PB samples were collected, and CD4‐ and CD8‐T subsets were analyzed through flow cytometry. Multiplex bead‐based LEGENDplex™ were used for cytokine quantification.

**Results:**

Higher CD8 percentages were observed in IPF than in HCs and PCLF (*p* = 0.020 and *p* = 0.007, respectively). PCLF subgroup showed higher Th‐naïve, Th‐effector, Tc‐naïve, and Tc‐reg percentages than IPF (*p* < 0.001; *p* = 0.018; *p* = 0.005; *p* = 0.017, respectively). Th‐naïve and Tc‐naïve inversely correlated with Tc‐reg (*p* < 0.0001, *r* = −0.61 and *p* = 0.005, *r* = −0.39, respectively). Tc‐naïve‐PD1 and Tc‐effector‐PD1 percentages were higher in PCLF than IPF (*p* < 0.001), while Tfh‐reg and Tfc‐reg were significantly higher in IPF than PCLF (*p* < 0.001). IL‐4, IL‐2, TNF‐α, and IL‐17A were more expressed in PCLF than IPF (*p* < 0.001). IL‐8 directly correlated with Tc‐naïve percentages in PCLF (*p* = 0.018, *r* = 0.35).

**Conclusion:**

A variety of immune cells is involved in the development and progression of pulmonary fibrosis confirming an immunological similarity between IPF and PCLF. T‐reg cells play a key role in the worsening of the disease. High cytokine values showed a pro‐fibrotic environment in PCLF patients, suggesting dysregulation of the immune system of these patients. Moreover, the immunological similarity between IPF and PCLF patients suggests that SARS‐CoV2 infection may trigger the activation of biological pathways common with IPF.

## Graphical abstract

A variety of immune cells are involved in the development and progression of pulmonary fibrosis confirming an immunological similarity between idiopathic pulmonary fibrosis and post‐COVID‐19 lung fibrosis (PCLF). T‐reg cells play a key role in the worsening of the disease. High cytokine values showed a pro‐fibrotic environment in PCLF patients, suggesting dysregulation of the immune system of these patients.

**Abbreviations**
CD: cluster of differentiation
COVID19: coronavirus disease 19
CXCR: CXC chemokine receptor
HRCT: high resolution computed tomography
IL: interleukin
ILD: interstitial lung disease
IPF: idiopathic pulmonary fibrosis
PCLF: post‐COVID‐19 pulmonary fibrosis
Tc: T cytotoxic lymphocytes
Tc‐reg: regulatory T cytotoxic lymphocytes
Tfc: T follicular lymphocyte cytotoxic
Tfh: T follicular lymphocyte helper
Th: T helper lymphocytes
Th‐reg: T helper lymphocytes regulatory

## 1 Introduction

According to the World Health Organization, a substantial percentage of patients affected by coronavirus disease 19 (COVID‐19) develop post‐acute complications leading to a significant burden in terms of morbidity and healthcare costs. Evidence suggests that COVID‐19 is associated with subacute and long‐term effects. Post‐COVID‐19 lung fibrosis (PCLF) is among the most dreadful complications as it is associated with significant morbidity and long‐term impact on respiratory health. Studies report that 33% of patients may show evidence of parenchymal fibrosis at a computed tomography (CT) scan 6 months after the resolution of acute illness [1]. Moreover, a set of conditions characterized by immune perturbations could determine the pathophysiology of post‐COVID‐19 [2]. Infection and inflammatory processes may compromise the lungs' architecture and local immune response activation. In this altered microenvironment, T cells, including various subsets, are recruited to the lung tissues and alveolar spaces, potentially promoting fibrotic processes. Conversely, other studies indicate that specific T cell subsets may exert regulatory functions that could potentially limit fibrosis onset and/or progression [3]. As reported in our previous study [4], the development of PCLF is associated with immunological abnormalities, somewhat similar to those observed in idiopathic pulmonary fibrosis (IPF). Strong T cell activation promotes the progression of both diseases (PCLF and IPF), with the unbalanced secretion of cytokines and chemokines being associated with the development and progression of fibrosis (IL‐4, TNF‐α, IL‐32, etc.) through the secretion of profibrotic cytokines and growth factors, facilitating collagen deposition and tissue remodeling. Besides CD4^+^ and CD8^+^ T cells, different T cell subsets act independently but interconnectedly, depending on the tissue microenvironment and stage of disease. T regulatory (Treg) cells play a critical role in immunological tolerance and balance [5] as they release cytokines such as IL‐10 and tumor growth factor‐β (TGF‐β), both involved in fibrogenesis. However, their role in pulmonary fibrosis pathogenesis may be pro‐ or antifibrotic, actually depending on the stage of the disease. An overactivation of such immune cells, associated with an exaggerated production of the above‐mentioned cytokines has been described in severe cases; it leads to cytokine storms that may contribute to the development or progression of fibrotic processes, sometimes dramatically, triggering acute exacerbation of disease. Concerning PCLF, this was reported for the first time by Bergantini et al. who showed that IL‐10, IL‐8, and IL‐32 serum concentrations were associated with fibrotic alterations of the lungs after COVID‐19 infection [6]. Indeed, the identification of patients with a higher risk of developing pulmonary fibrosis is essential to predict disease trajectories to timely intervention and treatment in post‐COVID‐19 patients. Numerous studies, including Kang et al. [7] and Baratella et al. [8], have explored the correlation between laboratory markers and radiological COVID scores at CT scans. Furthermore, Kang et al. [7] demonstrated the usefulness of laboratory markers, including lymphocyte count and IL‐6 measurement, associated with longitudinal CT imaging to predict and explain the change in lungs during and after the COVID‐19 infection.

Thus, in the present study, we further investigated the immunological dysregulations associated with PCLF through the analysis of different subsets of CD4‐ and CD8‐T cells in peripheral blood, providing a direct comparison with samples from IPF patients and healthy controls. The aim of the study was to identify the immunological similarities between PCLF and IPF patients to ensure better timely treatment.

## 2 Materials and Methods

### 2.1 Study Participants

A total of 100 patients, referred as outpatients to the Rare Lung Disease Center of Siena University Hospital, were enrolled in the study. Eight healthy subjects without lung disease or other comorbidities were included in the study as a control group. PCLF patients were recruited from a cohort of patients, previously hospitalized with COVID‐19 in the Siena COVID Unit, who agreed to participate in the post‐COVID‐19 follow‐up organized by Siena Hospital. The protocol envisaged a medical examination of patients 6 and 12 months after discharge. They underwent a thorough respiratory medical examination, including lung function tests (LFTs) with an assessment of the diffusion capacity of carbon monoxide (DLCO). According to the hospital protocol, high‐resolution computed tomography (HRCT) of the chest was performed as well. Patients with PCLF were identified according to the clinical and respiratory functional assessment and radiological features at the CT scan consistent with fibrotic interstitial alterations. Radiographic features suggesting possible development of pulmonary fibrosis included fibrotic inter‐ or intralobular thickening, associated or otherwise with air trapping and ground glass opacities. Otherwise, patients with a prior history of chronic obstructive pulmonary disease/asthma, malignancy, diabetes, heart disease, or renal failure, as well as those taking immunosuppressive or maintenance oral steroids, were excluded from the study.

Concerning the IPF subgroup, all patients enrolled were diagnosed and underwent follow‐up protocol in our center. The diagnosis of IPF was confirmed by a multidisciplinary team according to the international guidelines of the American Thoracic Society/European Respiratory Society (ATS/ERS) [9]. IPF patients were enrolled before the administration of any pharmacological treatment and displayed no signs of acute exacerbation and/or significant progression of disease.

**Figure 1:**
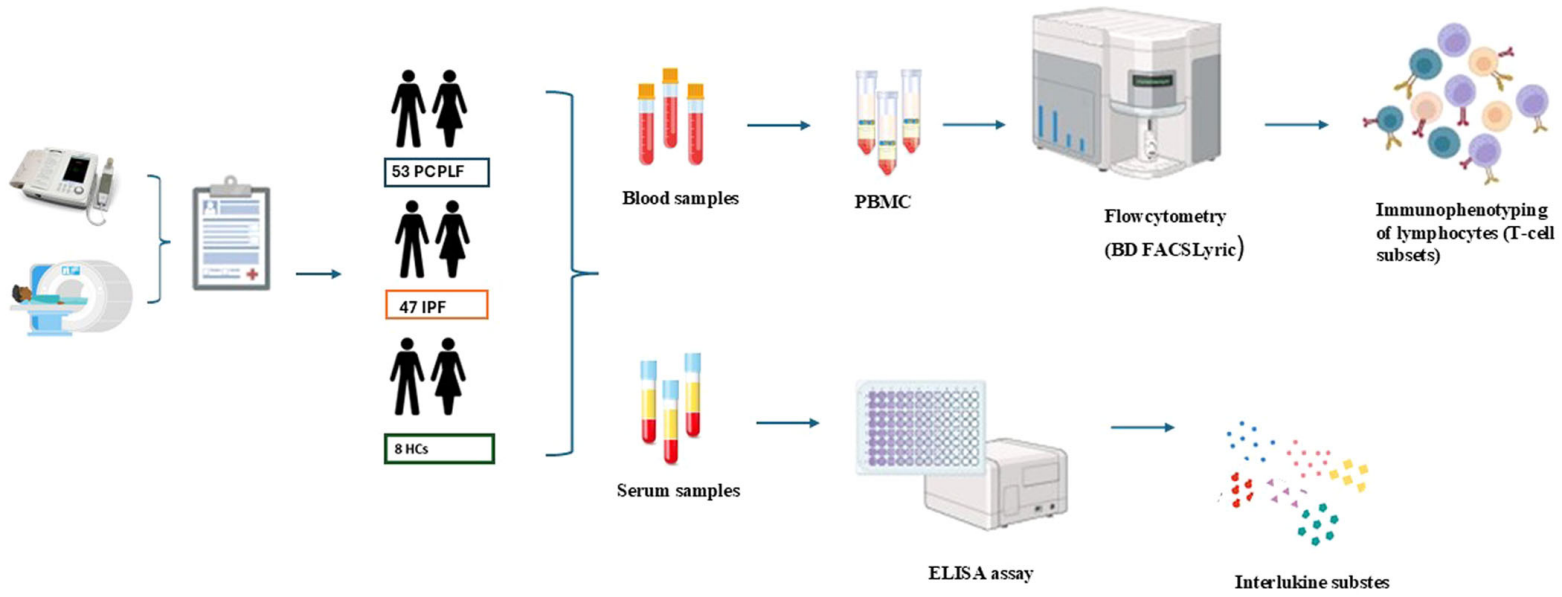
Flowchart of the study design from the enrollment of PCPF, IPF patients, and HCs for collection and processing of blood and serum samples to detect T cell subsets and interleukins through flow cytometry and ELISA assay. ELISA, enzyme‐linked immunosorbent assay; HCs, healthy controls; IPF, idiopathic pulmonary fibrosis; PCPF, post‐COVID‐19 pulmonary fibrosis.

The study design is reported in Figure 1.

All patients gave their written informed consent to participate in the study. The study was approved by the regional ethical review board of Siena, Italy (C.E.A.V.S.E. PAN_HUB_2021), and complied with the declaration of Helsinki. 2.3 Sample collection

Peripheral blood mononuclear cells (PBMC) were isolated from venous blood (BD Vacutainer EDTA tubes, BD Bio‐science, CA, USA) by density gradient centrifuging (Ficoll Histopaque‐1077, Sigma‐Aldrich, St. Louis, MO, USA) and cryopreserved for subsequent analysis (FBS + 10% DMSO). Serum samples were isolated from venous blood (BD Vacutainer Serum Tubes) by centrifuging at 1080*g* for 10 min and then stored at −80°C.

### 2.2 Flow Cytometry: Gating Strategy

**Figure 2:**
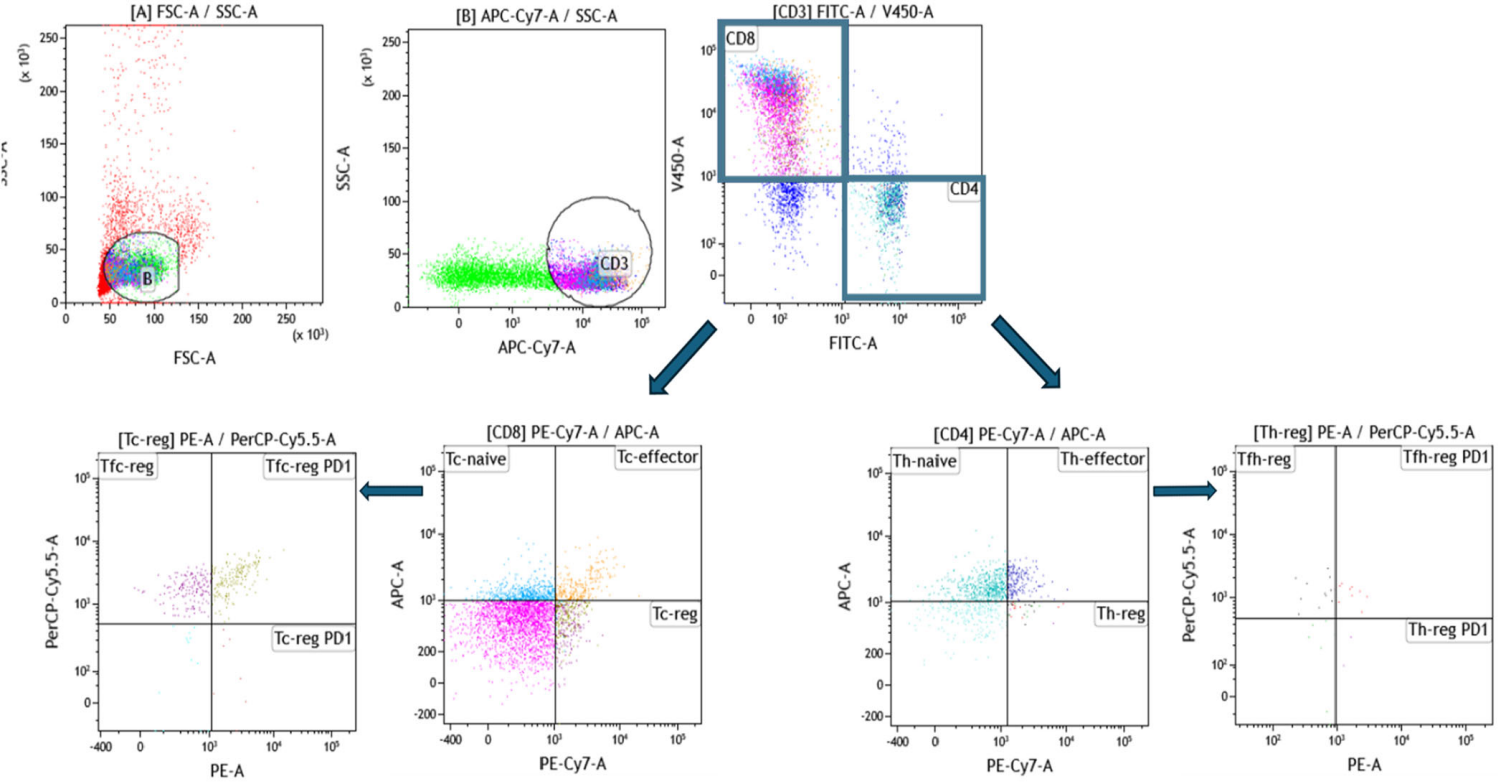
Lymphocytes were discriminated based on forward (FSC) versus side (SSC) scatters. Then, a dot plot was performed to identify CD4 from CD8 cells. Two dot plots were assessed on CD4‐ CD8 to discriminate Th‐ naïve; Th‐effector; Th‐reg; Tc‐ naïve; Tc‐effector; Tc‐reg cells. Using the PD1 marker, two dot plots were assessed on Th‐reg and Tc‐reg cells to distinguish Tfh‐reg; Tfh‐reg PD1, Th‐reg PD1; Tfc‐reg; Tfc‐reg PD1, Tc‐reg PD1 cells.

Cells stained with monoclonal antibodies (mAb) were analyzed by multicolor flow cytometry. Gating to determine major T cell types (CD4^+^ and CD8^+^) was performed using Kaluza Software 2.1 (Beckman Coulter, Brea, CA, USA) as shown in Figure 2. To further identify T helper and cytotoxic cell subtypes, we reclustered CD4 and CD8 T cells, obtaining 12 populations: Th‐ naïve (CD4^+^CD25^‐^CD127^+^), Th‐effector (CD4^+^CD25^+^CD127^+^), Th‐regulatory (CD4^+^CD25^+^CD127^‐^), T follicular helper‐reg (CD4^+^CXCR5^+^PD1^‐^), Tfh‐reg PD1 (CD4^+^CXCR5^+^PD1^+^), Th‐PD1 (CD4^+^CXCR5^‐^PD1^+^), Tc‐naïve (CD8^+^CD25^‐^CD127^+^), Tc‐effector (CD8^+^CD25^+^CD127^+^), Tc‐regulatory (CD8^+^CD25^+^CD127^‐^), Tfc‐naïve (CD8^+^CXCR5^+^PD1^‐^), Tfc‐reg PD1 (CD8^+^CXCR5^+^PD1^+^), and Tc‐reg PD1 (CD8^+^CXCR5^‐^PD1^+^). Multicolor flow cytometric analysis was performed using mAb (Table 1).

**Table 1:** The features of monoclonal antibodies used for multicolor flow cytometric analysis, including clone, fluorochrome, and company.

CD	Clone	Fluorochrome	Company
CD3	OKT3	APC‐Cy7	Biolegend
CD4	SK3	FITC	BD
CD8	SK1	BV421	Biolegend
PD‐1	PD1.3.1.3	PE	Miltenyi Biotec
CD127		APC	Biolegend
CD25		PE‐Cy7	BD
CXCR5		PerCP/Cy5.5	Biolegend

### 2.3 Cytokine Detection

The following cytokines were quantified by bead‐based multiplex LEGENDplex analysis (LEGENDplex Custom Human Assay, Biolegend, San Diego, CA, USA) according to the manufacturer's instructions: IL‐8, IL‐32, IL‐4, IL‐2, CXCL10 (IP‐10), IL‐1β, TNF‐α, CCL2 (MCP‐1), IL‐17A, IL‐6, IL‐10, IFN‐γ, and IL‐12p70. Reactions were run in duplicate using a BD FACSLyric flow cytometer (BD‐Biosciences San Jose, CA, USA). Data analysis was done using the LEGENDplex™ Data Analysis Software Suite (QOGNIT).

### 2.4 LFTs

The following lung function parameters were recorded according to standard ATS/ERS criteria [9] using a Jaeger body plethysmograph with correction for temperature and barometric pressure. We collected forced expiratory volume in the first second (FEV1), forced vital capacity (FVC), and diffusing capacity of the lung for carbon monoxide (DLCO). All parameters were collected in absolute values and as percentages of predicted values.

### 2.5 Statistical Analysis

Descriptive analysis was performed to evaluate medians and interquartile ranges or means ± standard deviations, as appropriate. A nonparametric one‐way analysis of variance (Kruskal–Wallis test) was used to compare the difference in lymphocyte subpopulations and cytokine concentrations between the two diseases and the control groups, determining if there was a significant difference between them. It compares the sum of ranks between the groups to assess whether the distributions of the groups differ significantly (*p*‐value less than 0.005). After the significant difference shown by the Kruskal–Wallis test, the Dunn Test was used as a post hoc test to identify which specific groups differ from each other. It calculates the rank sum differences between every pair of groups and uses a normal approximation to test if those differences are statistically significant (*p*‐value < 0.005). To detect correlations between immunological and clinical findings, the Spearman test was performed. It is a nonparametric measure of the strength and direction of the relationship between two variables. A *p*‐value less than 0.05 was considered statistically significant. Statistical analysis was performed using GraphPad 10.1.2 and Jamovi 2.3.21 software.

## 3 Results

### 3.1 Clinical Features of Patients With PCLF and IPF

Table 2 shows the demographic, clinical, and lung function parameters of patients with PCLF and IPF and HCs. To compare the immune profile of PCLF and IPF, we prospectively and consecutively enrolled 53 (median age 75 [69–80] years; 53% males; 94% never smokers) and 47 patients (median age 73 [68–78] years; 73% males; 58% never smokers), respectively.

**Table 2:** Clinical and demographic data of patients. The data are reported as median ± standard deviation.

	IPF (*n* = 47)	PCLF (*n* = 53)	Control (*n* = 8)
Sex (F/M)	13/35	23 F/29 M	4 F/4 M
Age (median)	73 ± 8.11	75 ± 8.24	69 ± 15.9
Smoking (never/former)	28/20^a^	52/3*	8/0
Lung function parameters (median ± standard deviation)			
FEV1%	76.5 ± 20.42	94 ± 16.07	
FVC%	73 ± 19.61	107 ± 10.98	
DLCO%	45.5 ± 33.26	60 ± 12.35	
Radiological findings	UIP (*n* = 48)	Fibrotic inter‐ or intralobular thickening (*n* = 55)/air trapping (*n* = 43)/ground glass opacities (*n* = 40)	

^a^ The IPF cohort showed a prevalence of smokers with respect to the PCLF cohort (*p* < 0.001).

When medical history was recorded, no PCLF patients had been vaccinated against SARS‐CoV‐2.

**Figure 3:**
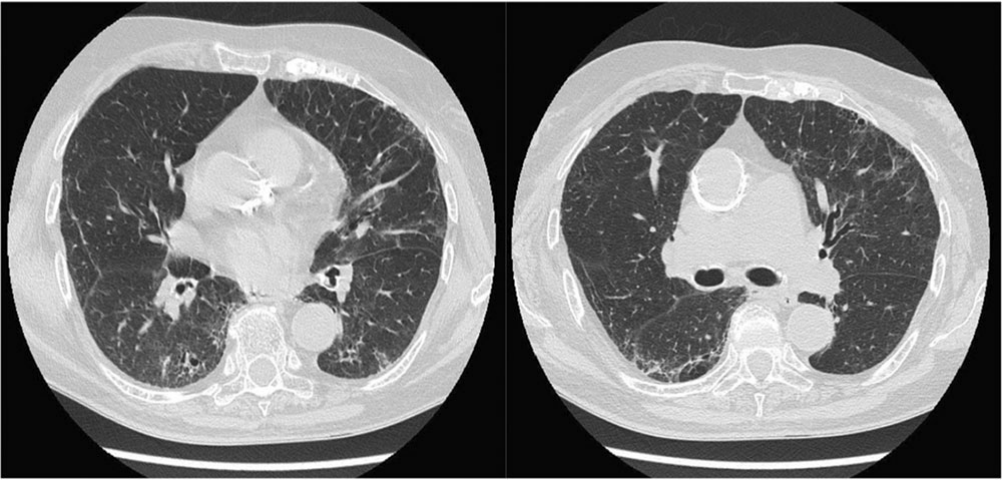
Axial HRCT images of a 79‐year‐old patient 12 months after hospital discharge, showing bilateral fibrotic reticular abnormalities associated with traction bronchiectasis. HRCT, high resolution computed tomography.

Figure 3 shows fibrotic reticular abnormalities in a CT scan obtained from a patient of our PCLF cohort 12 months after hospital discharge.

### 3.2 Immunological Differences Between PCLF and IPF Patients and Controls

No significant differences in CD4 T cell percentages were found between the two disease groups (PCLF and IPF), while IPF patients showed Higher CD8 T cells than HCs (*p* = 0.020) (Figure 4.1) and PCLF patients (*p* = 0.007). The imbalance in immune cells in IPF patients was also confirmed by an inverse correlation between CD4 and CD8 T cells (*p* < 0.0001; *r* = −0.746).

**Figure 4:**
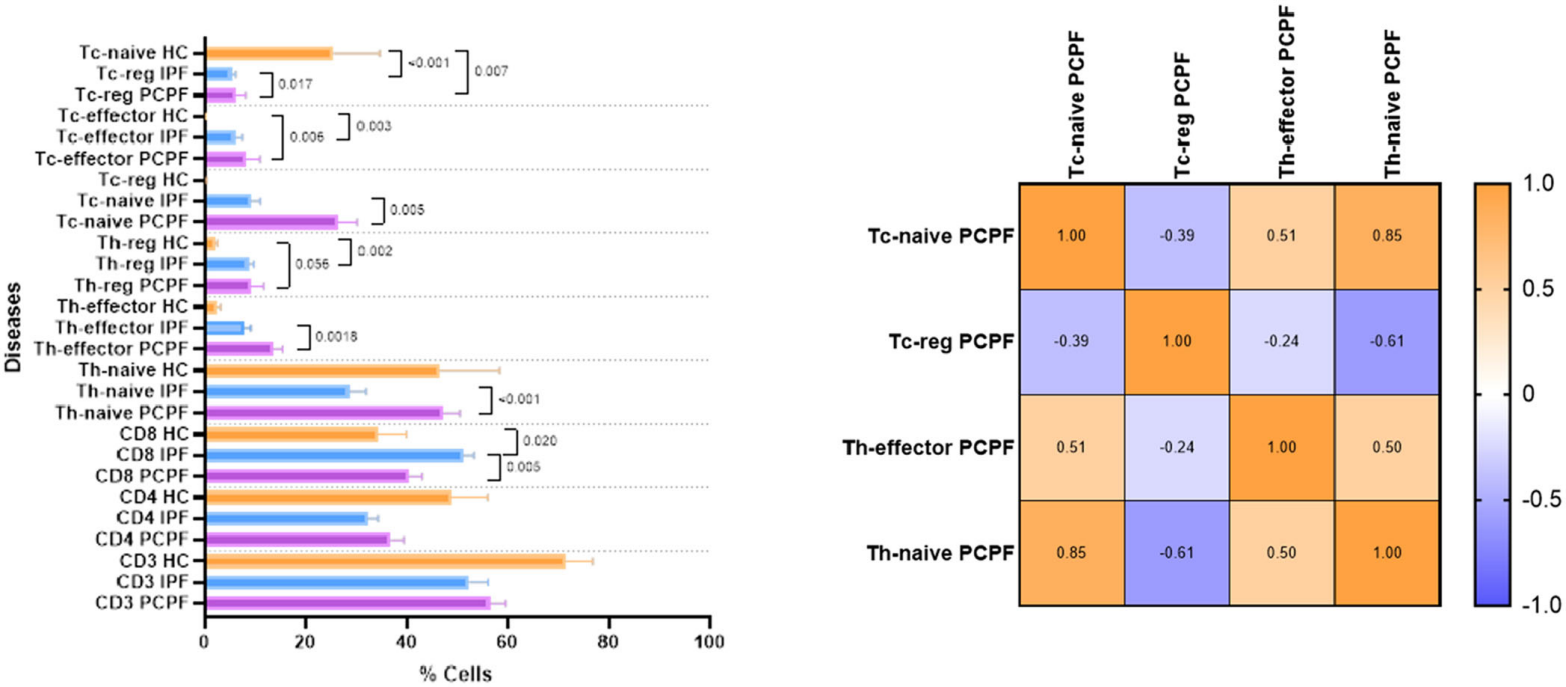
4.1 (left) Comparison of CD4‐, CD8‐, Th‐naïve, Th‐effector, Th‐reg, Tc‐naïve, Tc‐effector, and Tc‐reg cell percentages in IPF and PCPF patients and HCs: IPF, idiopathic pulmonary fibrosis; PCPF, post‐COVID‐19 pulmonary fibrosis. Numerical values in the figure indicate *p* values obtained by comparative analysis of PCPF, IPF, and HCs. 4.2 (right) Spearman correlation matrix of Tc‐naïve, Tc‐reg, Th‐effector, and Th‐naïve cell percentages in PCPF patients. The colors of the correlation matrix legend indicate rho coefficient values.

With regard to regulatory CD4 and CD8 cell subsets, PCLF patients showed higher Th‐naïve, Th‐effector, Tc‐naïve, and Tc‐reg percentages than IPF patients (*p* < 0.001; *p* = 0.018; *p* = 0.005; *p* = 0.017, respectively). Th‐naïve cell percentages were directly correlated with Th‐effector and Tc‐naïve cell (*p* = 0.0003, *r* = 0.50, *p* < 0.0001, *r* = 0.85), as well as Tc‐naïve and Th‐effector cell percentages (*p* = 0.0002, *r* = 0.51). Conversely, Th‐naïve and Tc‐naïve cell percentages were inversely correlated with Tc‐reg cell percentages (*p* < 0.0001, *r* = −0.61; *p* = 0.005, *r* = ‐0.39, respectively) (Figure 4.2). A significant increase in Th‐reg and Tc‐reg T cell percentages was detected in IPF patients with respect to HCs (*p* = 0.002; *p* < 0.001, respectively). Th‐reg, Tc‐reg, and Tc‐effector T cell percentages were also higher in PCLF patients than HCs (*p* = 0.056; *p* = 0.007; *p* = 0.006, respectively). These results highlight the important but controversial role that regulatory cells play in the fibrotic microenvironment.

### 3.3 Subsets of CXCR5‐Expressing Lymphocytes in PCLF and IPF Patients

Tc naïve PD1 and Tc‐naïve effector PD1 cell percentages were higher in patients with PCLF than IPF (*p* < 0.001), while Tfh‐reg and Tfc‐reg were significantly higher in patients with IPF than PCLF (*p* < 0.001). Conversely, Tfc‐reg cells expressing PD1 as well as Tfh‐naïve PD1 and Tfh‐effector PD1 cell percentages were higher in patients with PCLF than IPF (*p* = 0.028; *p* = 0.002; *p* = 0.009, respectively) (Figure 5.1). This exhausted cell subset could be typical of a the pro‐fibrogenic state. Moreover, Tc‐effector cells expressing PD1 were directly correlated with Tc‐reg PD1 and Tc‐naïve PD1 cell percentages (*p* < 0.0001, *r* = 0.62; *p* < 0.0001, *r* = 0.72, respectively), while percentages of Tfh‐naïve cells expressing PD1 were directly correlated with percentages of Tfh‐effector PD1 cells (*p* < 0.0001, *r* = 0.67) (Figure 5.2). Tfh‐effector and Tfc‐effector cell percentages were higher in patients with IPF than in those with PCLF. Tfc‐effector cell percentages were directly correlated with those of Tfc‐reg, Tfh‐reg, and Tfh‐effector cells (*p* < 0.0001, *r* = 0.76; *p* < 0.0001, *r* = 0.57; *p* < 0.0001, *r* = 0.58).

**Figure 5:**
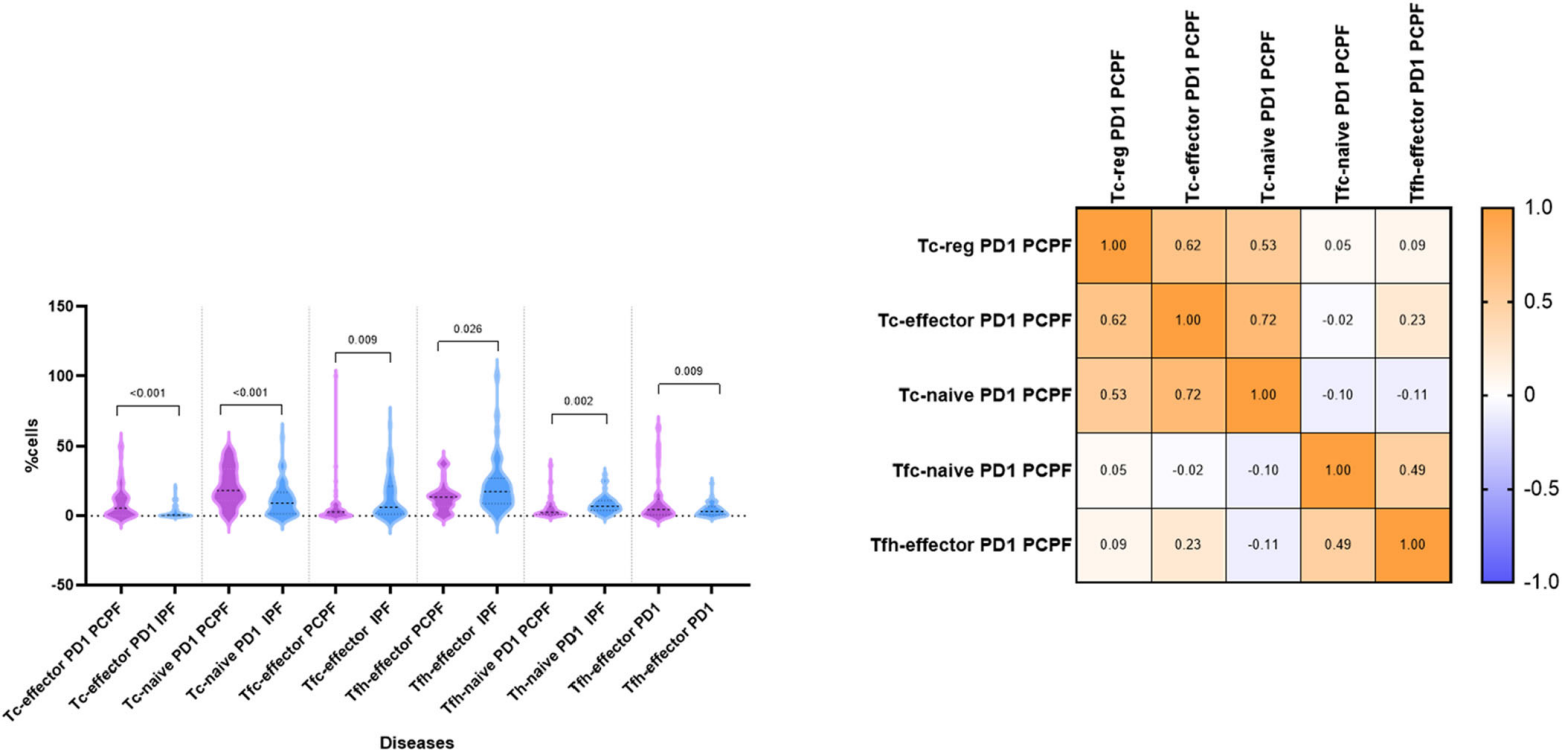
5.1 (left) Comparison of Tc‐effector PD1, Tc‐naïve PD1, Tfc‐effector, Tfh‐naïve PD1, and Tfh‐effector PD1 cell percentages in patients with IPF and PCPF (IPF, idiopathic pulmonary fibrosis; PCPF, post‐COVID‐19 pulmonary fibrosis). Numerical values in the figure indicate *p* values obtained by comparative analysis of groups: PCPF, IPF, and HCs. 5.2 (right) Spearman correlation matrix of Tc‐reg PD1, Tc‐effector PD1, Tc‐naïve PD1, and Tfh‐effector PD1 cell percentages in PCPF patients. The colors of the correlation matrix legend indicate rho coefficient values.

### 3.4 Cytokine Concentrations in PCLF and IPF Patients and Controls

Of the 12 cytokines analyzed, IL‐4, IL‐2, TNF‐α, and IL‐17A, all involved in the fibrotic process, were more highly expressed in patients with PCLF than in those with IPF (*p* < 0.001), whereas IP‐10 and MCP‐1 were expressed to a lesser degree (*p* < 0.001). Compared with HCs, PCLF patients expressed higher concentrations of IL‐2, TNF‐α, MCP‐1, and IL‐17 (*p* < 0.001).

**Figure 6:**
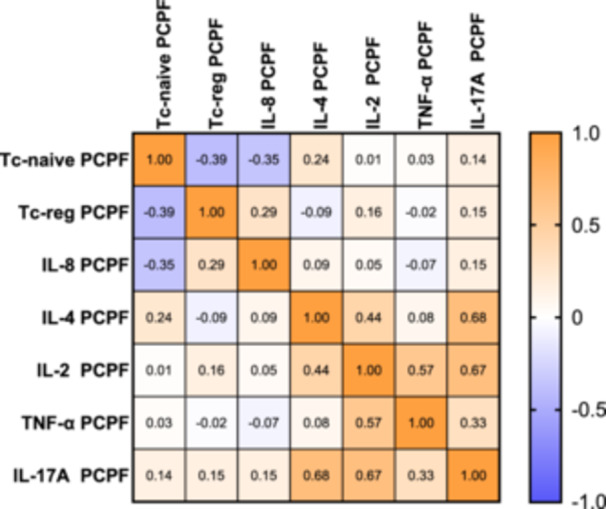
Spearman correlation matrix of cytokines analyzed in PCPF patients. The colors of the correlation matrix legend indicate rho coefficient values. The significant correlations were reported in the results section. IL, interleukin; PCPF, post‐COVID‐19 pulmonary fibrosis; Tc, T cytotoxic lymphocytes.

The matrix showed a direct correlation between cytokines in PCLF patients: IL‐4 was directly correlated with IL‐2 and IL‐17 (*p* = 0.002, *r* = 0.44; *p* < 0.0001 *r* = 0.68, respectively), IL‐2 was directly correlated with TNF‐α and IL‐17 (*p* < 0.0001 *r* = 0.57; *p* < 0.0001, *r* = 0.67, respectively), and TNF‐α and IL‐17 were directly correlated with each other (*p* = 0.027, *r* = 0.33). Moreover, IL‐8, typically expressed in lung fibrosis, was directly correlated with Tc‐naïve cell percentages in PCLF patients (*p* = 0.018, *r* = 0.35) (Figure 6).

## 4 Discussion

The aim of this study was to compare peripheral lymphocyte subsets and their specific cytokine expression patterns in groups of patients with PCLF and IPF, including also a small group of HCs as the control group.

Our findings supported the adaptive immune system dysregulation, previously described in IPF patients, due to loss of T cell tolerance leading to autoimmune responses that may be involved in the pathogenesis of such a disease. In line with literature data that reported IPF progression and severity of lung damage associated with increased percentages of CD8 cells, our IPF patients showed higher CD8 cell percentages than PCLF and HCs. The regulatory counterparts of Tc cells that expressed CD25 (IL‐2 receptor α‐chain) were lower in IPF than PCLF. CD25 is important in T cell proliferation, activation‐induced cell death, and the activity of regulatory (CD25^+^CD127^‐^) and effector (CD25^+^CD127^+^) T cells. Our PCLF patients showed high percentages of Tc‐reg and Th‐reg, similar to those of IPF patients compared to HC. However, Tc‐effector and Th‐effector tend to be highest in PCLF patients. Due to the high expression of CD25, T‐effector, and T‐reg cells are able to consume and limit the systemic concentration of IL‐2, ensuring the regulation of the immune balance. This finding was corroborated by the increased IL‐2 concentrations in PCLF patients than IPF and HC, supporting the importance of this cytokine in the early stages of infection, as well as the secondary adaptive response. T‐reg cells promote self‐tolerance and prevent excessive inflammation [10], which suggests that an imbalance of this subset might be involved in the development of lung fibrotic sequelae post‐COVID‐19.

A further cytokine mediator of lung fibrosis is IL‐8 which stimulates macrophages to migrate to fibroblastic foci via CXCR1/2 receptors [11]. As demonstrated by Bergantini et al. [6], patients affected by PCLF showed depressed serum concentrations of IL‐8, suggesting that exhaustion of the immune system drives long COVID. Our results first showed a direct correlation between IL‐8 and Tc‐naïve subsets. This correlation shows that a trace of the virus may trigger activation of T cells, and even interleukins, particularly IL‐8 in a fibrotic environment, suggesting that in PCLF patients' T cells are still tending toward a fibrotic phenotype.

Fibrotic processes in PCLF patients may also be suggested by the specific cytokine patterns expressed by T cells, resulting in higher concentrations of IL‐4, IL‐17, and TNF‐ α [11]. For instance, TNF‐α is a pleiotropic cytokine with a double role in the regulation of immune response, acting both as a pro‐inflammatory mediator and playing a vital role in the maintenance of immune homeostasis by limiting the extent and duration of inflammatory processes [12]. It is involved in the progression of IPF with increased TGF‐β1 production that stimulates fibroblast proliferation and induces collagen synthesis [13]. Our study showed greater expression of TGF‐β1 in PCLF patients than in IPF patients and HCs, demonstrating a fibrotic microenvironment after SARS‐CoV‐2 infection. IL‐4 and IL‐17 play a dual role in pulmonary fibrosis [11, 14, 15]. The former promotes the gene expression of collagen in lung fibroblasts promoting the differentiation of fibroblasts to myofibroblasts. The latter inhibits the autophagy of alveolar epithelial cells and collagen synthesis.

As a result of the defective protective function of Th‐reg cells in patients with chronic diseases, tissue tolerance is broken down, and ongoing immune responses do not decrease in a timely manner, as is the case in patients with inflammatory diseases.

Follicular regulatory T (Tfr) cells are recently defined as an effector subset of Tregs that express CXCR5. Tfr cells are involved in the generation and development of many immune‐related and inflammatory diseases. A recent study by d'Alessandro et al. revealed antifibrotic therapy preserved fibrosis progression stabilizing Tfh cells in IPF patients after 1 year of treatment, also confirmed by IL‐4 distribution in these patients.

Besides the upregulation of regulatory phenotypes, our PCLF patients also showed an exhausted T cell‐CXCR5 phenotype expressing PD1. The overexpression of this exhausted cell subset could reflect the pro‐fibrogenic state of PCLF patients, since PD1 has a role as a direct stimulator of TGF‐β, as already reported.

Our study showed some limitations. First, we had no clinical findings at the time of hospitalization including HRCT of the chest and LFT parameters to compare with those collected after at least 6 months of hospital discharge. Second, a longer follow‐up over than 12 months to correlate clinical and immunological findings.

## 5 Conclusion

The immune system is vast and complex, and a variety of immune cells are involved in the development and progression of pulmonary fibrosis. Although T‐reg cells have a controversial role in IPF, our results showed increased expression of CXCR5 and highlighted their involvement in the worsening of the disease. Regarding PCLF, our analysis of the T regulatory phenotype showed a depleted T‐CXCR5 phenotype reflecting the pro‐fibrogenic state of PCLF patients. These findings were also confirmed by increased cytokine concentration involved in the pro‐fibrotic environment, particularly IL‐4, IL‐17, and TNF‐α. Lastly, our findings displayed, even partial, immunological similarity between IPF and PCLF patients, suggesting that SARS‐Cov2 infection may trigger the activation of biological pathways common with IPF, therefore supporting the clinical indication of a strict follow‐up of patients with PCLF for an early detection of a progression of disease.

## Author Contributions

**Sara Gangi:** conceptualization, data curation, formal analysis, investigation, methodology, writing–original draft. **Laura Bergantini:** data curation, investigation, project administration, writing–original draft. **Irene Paggi:** investigation, methodology, writing–original draft. **Marco Spalletti:** methodology, writing–original draft. **Paolo Cameli:** data curation, investigation, methodology, writing–original draft. **Elena Bargagli:** project administration, writing–original draft. **Miriana d'Alessandro:** conceptualization, data curation, formal analysis, methodology, project administration, writing–original draft.

## Ethics Statement

The study was conducted in accordance with the Declaration of Helsinki and approved by the Local Ethics Committee of Siena University Hospital (C.E.A.V.S.E.) (protocol code PAN_HUB_2021).

## Consent

Informed consent was obtained from all subjects involved in the study.

## Conflicts of Interest

The authors declare no conflicts of interest.

## Data Availability

The data presented in this study are available on request from the corresponding author.
